# Reciprocity and Ethical Tuberculosis Treatment and Control

**DOI:** 10.1007/s11673-015-9691-z

**Published:** 2016-01-21

**Authors:** Diego S. Silva, Angus Dawson, Ross E.G. Upshur

**Affiliations:** Faculty of Health Sciences, Simon Fraser University, Blusson Hall, Room 11008, 8888 University Drive, Burnaby, B.C. V5A 1S6 Canada; Centre for Values, Ethics and the Law in Medicine, Sydney School of Public Health, University of Sydney, Level 1, Medical Foundation Building, K25, Sydney, NSW 2006 Australia; Dalla Lana Faculty of Public Health, University of Toronto, 155 College Street, Toronto, ON M5G 1L4 Canada

**Keywords:** Reciprocity, Tuberculosis, Ethics, Moral theory, Poverty

## Abstract

This paper explores the notion of reciprocity in the context of active pulmonary and laryngeal tuberculosis (TB) treatment and related control policies and practices. We seek to do three things: First, we sketch the background to contemporary global TB care and suggest that poverty is a key feature when considering the treatment of TB patients. We use two examples from TB care to explore the role of reciprocity: isolation and the use of novel TB drugs. Second, we explore alternative means of justifying the use of reciprocity through appeal to different moral and political theoretical traditions (i.e., virtue ethics, deontology, and consequentialism). We suggest that each theory can be used to provide reasons to take reciprocity seriously as an independent moral concept, despite any other differences. Third, we explore general meanings and uses of the concept of reciprocity, with the primary intention of demonstrating that it cannot be simply reduced to other more frequently invoked moral concepts such as beneficence or justice. We argue that reciprocity can function as a mid-level principle in public health, and generally, captures a core social obligation arising once an individual or group is burdened as a result of acting for the benefit of others (even if they derive a benefit themselves). We conclude that while more needs to be explored in relation to the theoretical justification and application of reciprocity, sufficient arguments can be made for it to be taken more seriously as a key principle within public health ethics and bioethics more generally.

To date, there has been little, although increasing, discussion of the role that the concept of reciprocity might play in justifying and legitimizing action regarding persons with infectious diseases, such as tuberculosis (TB), particularly those suffering from active pulmonary and laryngeal TB (Holm 2009; Viens, Bensimon, and Upshur 2009; Coleman et al. 2010; Silva and Viens 2015). Where this has happened, it has been done without reference to, or grounding in, any specific moral or political theories. Most discussions of reciprocity in the public health ethics literature has focused on the very specific issue of what is owed to persons subjected to isolation orders in terms of either (a) the material and psychological supports necessary to successfully remain in isolation, or (b) compensation for loss of material goods as a result of being in isolation. Few, if any accounts, have argued for compensation for the loss of freedom of movement necessary for isolation irrespective of a loss of goods or material burdens actually accrued. Even fewer discussions take into account any prior inequalities that may, in fact, have significantly contributed to the risk of developing the disease in the first place.

How should we conceptualize reciprocity and what is the scope of the obligation it imposes? In this paper, we argue that grounding the concept of reciprocity in specific moral and political theories can help clarify both the concept of reciprocity and the implications of the accepted obligation in relation to those with infectious diseases like TB. In Section One, we set out important background issues in relation to the causes and treatment of TB that will feature in our discussion. In Section Two, we describe, compare, and apply accounts of reciprocity drawing from Lawrence Becker’s virtue ethics version and Arthur Ripstein’s Kantian interpretation of reciprocity. We have been unable to find a clear and explicitly consequentialist account of reciprocity in the literature, so we have attempted to construct such a perspective. We seek to understand reciprocity in the context of these specific theories through their application to TB treatment and control. In Section Three, we build upon the existing literature about reciprocity, explore in more detail what reciprocity is and how it relates to other moral concepts, and offer our tentative suggestions for how we ought to think of reciprocity as a mid-level principle and its applications in the case of TB treatment.

It should be noted at the outset that we will remain neutral as to which theoretical backing for reciprocity should be ultimately adopted for two reasons. First, most of this paper needs to be dedicated to the exegesis of the theories and how they would apply to the case of TB. As such, the goal of this paper is to describe the accounts of reciprocity and to see how these versions could help elucidate our thinking about both reciprocity and TB care. Second, the application of all three accounts of reciprocity to the case of TB results in very similar conclusions, albeit through different arguments. In other words, the role of reciprocity as part of our decision making about, and justification for, TB care is ethically or politically overdetermined, and theoretical arguments about rival accounts can be postponed to another occasion.

Before we begin, a few definitions and distinctions are necessary. First, the term *politics* and its derivatives are held to denote those norms that are generally upheld by the state, state coercion, and by which citizens and residents must abide, whereas *ethics* or *morality* and their derivatives denote how people ought to behave regardless of whether the state would enforce such actions.^1^ For the purposes of this paper, in relation to societies’ and governments’ rights, obligations, and duties toward persons with TB (and vice versa), it does not matter too much whether the reader believes that there is a distinction between politics and ethics, or whether one assumes that ethics encompasses politics, etc. However, the distinction does matter for those who appeal to Kantian arguments since on such views moral ideas, like the oft cited categorical imperative, are unenforceable and hold little or no sway in the realm of politics, i.e., what can be justly enforced. Second, there exist questions about how exactly to describe reciprocity, i.e., is it a *virtue* and thus, a fundamental part of what ought to be part of our character, as Becker maintains, or is it a *concept* as used by Ripstein, or is it a moral value, consideration, or principle? For the purposes of this paper, we will ultimately settle on using reciprocity as a mid-level, action-guiding principle, but only after arguing that its use in public health ethics and in the context of TB is theoretically overdetermined.

## Section 1: Current State of Tuberculosis Treatment and Control

Understanding the context of tuberculosis as a disease is vital to understanding why reciprocity is relevant to discussions of ethical TB treatment and control. TB is a bacterial disease that kills approximately one million people per year. However, we are not all at equal risk. TB has been described as a “disease of poverty” and it can be seen as a failure of society to care for those of lower socioeconomic status (SES) (Uplekar et al. 2006). Its mode of transmission and disease aetiology (whereby transmission is exacerbated by extended and close contact with persons in settings with little sunlight and air circulation and by co-morbidities that reduce immunity) ensures that the deaths caused by TB are disproportionately found in persons of lower SES, including further marginalized and disadvantaged populations such as indigenous peoples, prisoners, and migrants. This unequal distribution of the burden of TB is not solely seen at a global level, where Asia and Africa account for almost 90 per cent of cases, but also within high-income countries (World Health Organization 2014). For example, in Canada, Aboriginal peoples represent just 3 per cent of the total Canadian population, but accounted for almost 23 per cent of the cases of TB in 2011 (Public Health Agency of Canada 2014).

Tuberculosis care consists both of clinical interventions and public health measures, the latter intended not necessarily for the benefit of the patient but to protect those who are currently uninfected. The main public health function once someone has TB consists of isolation and deployment of directly observed therapy (DOT). Isolation may be limited to separating the infected person from others, including family members, through use of a mask. This may be appropriate in cases where the level of a patient’s infectivity is low and the patient adheres to mask-wearing. However, in other cases, for example where a patient chooses repeatedly to put others at risk through their behaviour, isolation may involve court-ordered restrictions including detention in hospitals. In the extreme, particularly in cases of drug-resistant strains of TB, where therapeutic options have been exhausted, we may face a situation where a patient needs to be in complete physical isolation for the rest of their lives. It is important to note that isolation has no clinical benefit to a TB patient. In contrast, DOT, which consists of a public health worker physically watching a TB patient swallow their medications, is generally understood as having both a clinical and public health function. As a clinical measure, the public health official is responsible for observing for potential side-effects stemming from TB drugs and for providing a patient with emotional support. TB patients need someone to monitor drug use due to the many serious potential side-effects that may result from first-line drugs, including hepatoxicity and temporary deafness. When coupled with the length of the average course of drug treatment for TB, which can be from about six to twenty-four months, the side-effects of medication are a significant cause of patients not finishing their course of treatment, which has been a leading cause of the rise of multi- or extensively drug resistant TB (M/XDR-TB). As a public health measure, DOT tries to stem the spread of TB and M/XDR-TB. It is important to note, therefore, that persons of lower SES are not only at higher risk of contracting the disease, but, as a result, also carry a disproportionate burden in relation to the potential harms of TB treatment and control. TB patients are often doubly disadvantaged by the consequences of the treatment regimen itself. For example, in South Africa people undergoing tuberculosis therapy will lose their welfare benefits while hospitalized, while said benefits may be the sole means of revenue for many families (Singh, Upshur, and Padayatchi 2007).

The advent of M/XDR-TB raises stark and difficult ethical issues. Treatment is prolonged, with significant side effects, and outcomes often remain very poor with approximately 50 per cent mortality rate (World Health Organization 2014). Steadily increasing rates of drug resistance raises the spectre of cohorts of patients still capable of transmitting tuberculosis. All such transmissions will lead to growing numbers with latent infection that will be drug resistant when it manifests as disease. As well, there may be a time when complete drug resistance emerges, leaving very few therapeutic options available. Isolation may well be the only option available to prevent further spread of disease.

Part of society’s failure toward persons of lower SES in the treatment of TB is that until recently there had been no new TB drugs in over forty years. Like many other diseases that affect persons of lower SES, research and development into new TB drugs has been slow to materialize. However, in 2012−2013, two new drugs were given conditional approval for use in combating M/XDR-TB: bedaquiline and delamanid. First, bedaquiline was approved by the U.S. Food and Drug Administration (FDA) in December 2012. Although the drug apparently helps reduce the amount of bacteria in a patient’s sputum leading to improved cure rates, in the main study of the drug 11.4 per cent of those in the treatment arm died, while only 2.5 per cent of those in the placebo control arm died and this “imbalance in deaths is unexplained” (Food and Drug Administration 2012). The World Health Organization’s (WHO) expert group found the evidence in support of bedaquiline to be of “very low” quality (World Health Organization 2013). Second, delamanid was approved by the European Medicines Agency (EMA) in November 2013 to be used in combination with other drugs in the treatment of MDR-TB, but stated it be used “only when alternative medicines cannot be used in its place” (European Medicines Agency 2013, ¶4). One unaccounted issue with delamanid is that the treatment group in the main study suffered from prolonged QT intervals (i.e., a measure of how healthy the heart is, where longer intervals signal increased susceptibly to heart attacks) at a higher rate (13.1 per cent at 200mg) than the placebo group (3.8 per cent at 200 mg), though no deaths were associated with the prolongation (Gler et al. 2012). With regard to both bedaquiline and delamanid, the WHO, FDA, and EMA have all called for stringent pharmacovigilance strategies to evaluate the long-term effects of these new drugs (World Health Organization 2013; European Medicines Agency 2013; Food and Drug Administration 2012). These new pharmaceuticals are, at best, less than desirable solutions to the clinical treatment of TB, and do not seem to radically shift the need for isolation and DOT as primary public health measures. They also raise problematic questions about the balance of preventive versus curative approaches; arguably, emphasis on the latter has been responsible for the creation of drug resistance in the first place.

As a preliminary definition, reciprocity denotes returning goods in proportion to those received and compensating those we have harmed. We see reciprocity as being of key importance in addressing the inequalities and redressing some of the harms associated with TB. First, the social and political nature of TB provides an opportunity to use reciprocity to argue that societies and governments might have an obligation to persons of lower SES to remove, or at least reduce, the unequal risk factors associated with contracting TB. Second, reciprocity can be used to explore exactly what obligations follow in cases of isolation. Third, reciprocity can potentially be used to temper any pressure that societies or governments may level against persons with M/XDR-TB to use novel TB drugs that have only received provisional regulatory approval and may have side effects that are serious and/or not fully accounted for. Such uses of reciprocity go far beyond a simple compensatory model of paying back direct losses that is often at the heart of existing discussions of reciprocity in relation to public health cases. So, how could we justifiably use reciprocity through an appeal to normative moral theory?

## Section 2: Three Accounts of Reciprocity

### A Virtue Ethics Approach to Reciprocity and Tuberculosis

Perhaps the best known articulation of reciprocity in traditional moral philosophy is that proposed by Lawrence Becker (1986). In his view reciprocity is the disposition “to return good in proportion to the good we receive, and to make reparations for the harm we have done” (Becker 1986, 3) while resisting evil but never returning evil-for-evil. Becker’s version of reciprocity is grounded in virtue ethics. In particular, Becker is concerned with articulating those moral considerations that will foster a positive moral disposition, toward us and others, since morality is fundamentally about relationships between people. On his view, reciprocity is a good thing insofar as it helps build bonds between people and helps boost a person’s sense of self-worth, both of which are important for the flourishing of human beings. Obligations of reciprocity exist regardless of whether or not we have asked for the good we have received, since reciprocating good gives human life greater predictability and better social cohesion. According to Becker, this does not entail that people are justified in meddling in other people’s lives, even if there are good intentions behind the meddlesome acts; rather the loss endured (e.g., some reduction of privacy) negates the need to reciprocate in these cases.

Reciprocity for Becker consists of three main aspects. First, we ought to return good for good received. Doing so gives humans “pleasure” and “generates many of the conditions under which the sustained pleasure of social relationships are possible,” while “sustaining mutually advantageous exchanges” (Becker 1986, 90). Reciprocating, moreover, is morally required to be proportionate to the good received, first and foremost, or relative to the sacrifice it would take to return a good received. Reciprocity is to be promoted as a disposition because of the good that it promotes in social relationships. It is necessary to be as commensurate as possible in returning benefits, although if the cost in returning the benefit is so high as to damage the person who must reciprocate, or if it is impossible to reciprocate (e.g., receiving a kidney donation), then returns need not be exactly proportionate. To illustrate this point, Becker gives the example of a mother with a high income giving her child a bike, which becomes the child’s favourite toy and a memento for life. The mother’s sacrifice is small relative to the benefit the child receives; yet the child cannot, at least immediately, provide the mother with a comparable benefit, either at all or at a very high cost to the child. The child may, later in life, care for the mother when the mother is elderly and unable to care for herself, perhaps. Regardless, the point is that the child does not have an obligation to reciprocate his mother’s gift in the exact same manner or amount since doing so will place a grave burden on the child.

Second, since the goal for Becker is to establish reciprocity as a moral consideration that promotes social relationships, it is also imperative that while evil may be resisted, it must not be returned in kind. Although it may feel good to exact revenge on an enemy, reciprocity does not permit revenge since doing so would be to endorse a bad disposition for humans, one that would not result in better functioning of the species. That does not mean that wrong-doers should go unpunished for their deeds but that punishment ought not to take the form of revenge, but rather, as is currently the case, should be the purview of the state and the legal system, including the criminal justice system for more extreme matters.

The third and final aspect of reciprocity is that we should act to restore dealings with those to whom we have done wrong, as justified again by the argument that doing so best promotes—and in this particular case, helps repair—social relationships. We must restore the person to the place they were prior to our wronging them, as best as possible, although there are many cases when it is impossible to actually restore the person to their original situation. The practice of restitution “does help to restore the confidence required for free and reciprocal exchanges” (Becker 1986, 102).

Of particular importance to considerations involving public health is Becker’s discussion of *corporately produced goods*, including the good derived from public goods, which raises the problem of “what sort of reciprocation to make when we seem unable to do anything that equals either the benefits we have received or the sum total of the sacrifices that have gone into producing those benefits” (Becker 1986, 113). In other words, we, as individuals, can do nothing *qua* individuals to reciprocate the benefits received or the cost it takes to provide said benefits. Take the classic example of water fluoridation: the benefit to each of us is immense, and the cost to the various levels of governments to provide fluoridated water presumably high, so there is no way that we can return the level of benefit or sacrifice that the whole has provided each member of the group individually. Becker suggests that since:

Such benefits typically come to us by way of people’s participation in on-going social institutions. … What is *fitting* is reciprocal participation in those institutions. … A proportional return is a “proportional” level of participation. And since (by hypothesis) one cannot match the benefits received, one can only take the second-best option and make an equal sacrifice. But equal to what? Equal, evidently, to the sacrifices made by others who have comparable abilities and resources, and who have benefited in a comparable way (Becker 1986, 114, *emphasis original*).

Stated simply, we can only do our small part to contribute to the good and proper functioning of the social institutions in question. To return to the case of water fluoridation, our paying of taxes might be a simple and straightforward way in which we reciprocate the good we receive from such a public health measure. Presumably for Becker, the government ought to coordinate such measures, such that it distributes the goods of social institutions based on the sacrifices of the many as a group, not on an individual basis.

Since for Becker there are other moral considerations that help create positive moral dispositions beyond reciprocity, it is important to resist the temptation to use the idea beyond its intended purposes. However, the idea of reciprocity may still help us make sense of the obligations of persons with tuberculosis and society’s response to the isolation of these patients. First, Becker’s theory provides us with the most straightforward account of why we, as a society, need to provide for, support, and compensate those TB patients who abide by isolation orders as a public health intervention through the idea of reciprocity. Individuals who abide by isolation orders are conferring a good to the public at a high level of sacrifice to themselves. However, a simple compensation model of reciprocity seems inadequate for those patients detained in isolation until their death, as may be the case with those suffering from incurable cases of M/XDR-TB. Their sacrifice is not a mere temporary inconvenience, and society may owe a great deal to them, through the broader account of reciprocity we are arguing for in this paper. Making sure that a person does not languish while in isolation seems to be a fitting and straightforward example of how we can, as individuals and as a collective, most likely discharging our actions through government agents, can reciprocate for the good we receive from others being in isolation for TB. Simply stated, we have a moral obligation to support and compensate those persons who abide by isolation orders for the good they confer to society at a great cost to themselves, regardless of the duration of isolation.

Second, since a TB patient presumably benefits from social institutions, their abiding by isolation orders may be an instantiation of reciprocating the good they have received from the institution of public health as a whole. However, given that those most likely affected by TB are persons of lower SES, it seems unlikely that we can justify the need for restrictive measures, such as isolation, through reciprocity on the part of individuals. For Becker, it is likely that given the reality of the TB context, isolation ought to be maintained for other important normative reasons (e.g., minimizing risk of harm to others) not on the grounds of reciprocity.

Finally, assume for the sake of argument that the counterfactual were true, and that all persons have a relatively equal chance of contracting TB and developing the disease, such that reciprocity arguments could be used to justify isolation orders—it does not necessarily follow that such arguments can also be used to justify forcing TB patients to take new anti-tubercular medications that have potentially dangerous side effects. Recall that for Becker, we must return good in a proportionate amount to the good we have received, first and foremost, but not if the level of sacrifice required is so high as to permanently cripple us and our ability to live our future lives. So although one could present a reciprocity argument for justifying why TB patients need to abide by isolation orders in order to return to the community corporately produced goods, the same argument would not seem to extend to being required to take bedaquiline and delamanid for resistant strains of TB given the level of risk these anti-tubercular drugs impose on individuals.

### A Kantian Account of Reciprocity

So far as we know, Kant never wrote about tuberculosis. Taking this to heart, we want to present a Kantian approach to reciprocity which does not turn on whether or not harms occur but rather speaks to the very nature of human freedom and justice, such that it elucidates how a Kantian might think about TB care. Regardless of whether it is ultimately sound, an argument that speaks of reciprocity without reference to harm and benefits is rare. Holm notes that “a question concerning whether a person should be compensated for a harmless liberty restriction imposed by the state” is interesting but can be ignored since “most cases of public health detention involves harmful liberty restrictions” (Holm 2009, 200). As we will argue shortly, this need not always be the case. Moreover, considering reciprocity as it relates to the form of freedom may speak to justice issues within the broader context of TB and provide a compensatory and distributive justice argument based on reciprocity.

According to Kant there exists an innate right of freedom: “[f]reedom (independence from being constrained by another’s choice), insofar as it can coexist with the freedom of every other in accordance with a universal law, is the only original right belonging to every man by virtue of his humanity” (Kant 1996, 6:237). As with most things relating to Kant, it would take a lot of space and time to properly analyse this passage, which serves as the foundation of the rest of his arguments in the political realm. For our purposes, we will concentrate on some key points to demonstrate how a Kantian might approach TB treatment and control.

Kant describes freedom as a quality that human beings have to be their own “masters” (1996, 6:238) and one that applies equally to all persons, i.e., “independence from being bound by others to no more than one can in turn bind them” (1996, 6:237). Equal freedom is something that people have (or being equally free is something that people are); it is a *quality* of being human, irrespective of what can be *achieved* with that freedom (Ripstein 2009). As Ripstein notes, equal freedom

is not a matter of people having equal amounts of some benefit, however to be measured, but of the respective independence of persons from each other … a system of equal freedom is one in which each person is free to use his or her own powers, individually or cooperatively, to set his or her own purposes, and no one is allowed to compel others to use their powers in a way designed to advance or accommodate any other person’s purposes (Ripstein 2009, 33).

Each person, then, has the freedom to independently choose to set goals and purposes and go about attaining them. Being a “master” of oneself, in turn, means that one’s ability to set and attain goals and purposes is not at the discretion or use of another person. This understanding of freedom, however, is only compatible with the freedom of others to set their own goals and purposes in life; thus, this understanding of equal freedom is protected and promoted only if each person reciprocates by acting in such a manner as to promote the freedom of others. The innate right of freedom does not entail, however, that merely frustrating an individual’s achievement of a goal or obtaining a good constitutes an infringement on his or her freedom. For example, a mutually exclusive competition for a resource between *A* and *B* suggests that if *A* obtains the resource, *B* will not, and vice versa; this is morally permissible and to be expected. However, if *A* somehow removes *B’s* ability to obtain a particular resource (e.g., through threat of force), then *A* wrongs *B*. To not allow competition runs contrary to Kant’s notion of equal freedom, since “[t]o insulate one person from all the effects of the choices of others would subordinate everyone else to that person’s choice” (Ripstein 2009, 39).

Kant’s position on restricting freedoms—or coercion, to use his language—is already contained in the postulate of freedom itself. For Kant, under a reciprocal understanding of freedom between all individuals, a hindrance on said freedom is a wrong, but a “hindering of a hindrance to freedom” is right (Kant 1996, 6:231), i.e., *B* or *C* can hinder *A’s* freedom if *A* is hindering *B’s* freedom. A straight-forward example is the case of self-defence: if *A* is about to assault, or is assaulting, *B*, then *B* has a right to defend him- or herself and *C* (a third party) also has a right to defend *B*, even if doing so constitutes a hindrance on *A’s* ability to assault (since *A* would not have a right to assault in the first place). As such, coercion is an extension of the idea of freedom as equal and reciprocal among individuals since coercion is seen as a particular instance of a limitation or interference toward actions that one would not have a right to commit in the first place (e.g., assault).

An ensuing and correlative question for Kant’s account of restrictions or coercion by the state is to ask when the state can use its powers to hold a person responsible for their actions. According to Ripstein,

whether a person is accountable depends on what that person owes to others … it articulates the nature of responsibility in terms of reciprocal norms of conduct, that is, norms governing what people owe to each other within personal interactions … the reciprocity conception does not, by itself, specify the content of those norms concerning what is owed very fully (Ripstein 2004, 371).

In other words, instead of being responsible for the good or bad that befalls another person, responsibility is established, in a formal manner, on the basis of norms of conduct, that is, on the basis of what types of conduct the state ought to enforce between persons. Recall the example above, regarding competition for limited resources; although *A* may be causally responsible for *B* not getting a particular resource, and although that may result in hardship for *B*, *A* has done nothing wrong, so long as *B* had an equal chance to obtain the same resource. At its foundation, the type of conduct that can be enforced by the state is that which promotes self-mastery or self-determination equally among a population. Nothing has yet been stated as to the content of this formal sense of reciprocal freedom and responsibility. However, this Kantian position should not be understood as rejecting the redistribution of material resources. On the contrary, Kant has an argument for redistribution that follows from the postulate of freedom (setting aside whether or not the argument follows), namely, that to be self-determined one needs a certain baseline amount and certain kinds of material goods, otherwise some people become dependent upon others for their survival and freedom (Kant 1996, 6:326). Stated in terms of responsibility, a certain threshold of material security is necessary to be held responsible for one’s actions.

A plausible Kantian justification for isolation for TB, then, would be grounded in reciprocal norms of conduct, namely, doing one’s best to avoid infecting others with TB whereby that norm is applied to everyone equally (e.g., Bill Gates would have to remain in respiratory isolation, too). Being in isolation would clearly restrict, at least temporarily, one’s ability to make certain life choices, but does not affect, and should not affect, one’s freedom to determine the path of one’s life in any meaningful way over the course of a person’s whole life. In other words, isolation for TB control, which requires restrictions on freedom of movement, is just so as to avoid disease transmission and, as a norm, ought to apply to everyone equally, but any more than what is required to protect and promote the public’s health is unjust. The difficult case from the Kantian perspective might be those instances of incurable M/XDR-TB where a person must remain in isolation until they die. Here it seems that a Kantian might answer that given the risk that a such an individual might pose upon others in his or her community, at least their freedom of movement must be removed permanently so as to protect the freedom of others, though then the reciprocal obligation to care for such an individual would be equally high, relative to that individual’s sacrifice.

In fact, caring for all TB patients in isolation, regardless of duration, should not be regarded as an act of charity, rather any TB patient in isolation is entitled to basic life necessities since that is what is necessary so that he or she is not beholden to others, thereby maintaining, as best as possible, their ability of self-mastery. The need to support those persons in isolation with TB should be extended to everyone, even if the TB patient has enough resources to survive (e.g., has enough money to order healthy takeout while in isolation). The state should offer even the rich, even Bill Gates himself, the basic life resources necessary to survive, should they contract TB. It may be morally nice of persons who are monetarily rich to forgo such resources, but the offer must be there so as to uphold an equal sense of freedom.

There seem to be at least two more relevant conclusions that we can draw from a Kantian account of reciprocity for TB care, beyond the issue of isolation. First, on this view one should not be forced to accept public health measures that could permanently and irrevocably harm him or her, such that they would be left in a position whereby their capacity for freedom is permanently damaged, especially in cases where there exist other public health measures to arrest the spread of TB.^2^ For example, it is unclear that someone suffering from M/XDR-TB, who is fully compliant with isolation orders, ought be compelled to take bedaquiline or delamanid, even if either is clinically indicated, given the side effects or adverse reactions for both. Second, a reciprocal notion of equal freedom would also seem to support the need to fix the social and economic conditions that lead to the unequal distribution of burdens associated with TB, including but not limited to isolation, in the first place. The living conditions of many TB patients are abhorrent and exist due to actions or inactions of persons of wealth or in positions of power, such that these conditions provide an unequal access to goods, private and public, including healthcare and basic life necessities (e.g., water, food, etc.) that increase the likelihood of contracting TB. Such conditions are not examples of merely allowing competition for resources amongst equals but include the systematic control of some by others. The case of mining companies in South Africa, whereby miners are susceptible to TB transmission, or Russian prisoners not receiving adequate TB care are examples where the power imbalance are not merely that of competition over resources but places one group of persons subservient to another group of people so as to warp the very structure of equal freedom itself.

### A Consequentialist Account of Reciprocity

Many accounts of consequentialism are simplistic. Consequentialism has moved on since the death of Bentham and the group of theoretical positions falling under this name is more robust that often thought. By consequentialism we mean any normative account that focuses on the outcomes (i.e., consequences) of actions. Such theories may include those focused on utility, or other possible goods such as equity, or even a plural group of goods. Consequentialists will disagree amongst themselves about what the relevant good or goods might be. There may also be disagreement about whether the relevant good or goods ought to be maximized.

In the absence of an individual writer with a robust consequentialist account of reciprocity, we will seek to reconstruct such a position here. Such a view might see reciprocity as either being intrinsically valuable (i.e., valuable in itself) or as being extrinsically valuable (i.e., valuable for the end that is produced). An intrinsic account may hold reciprocity as being either the good that we ought to use to assess actions (perhaps even maximize) or as being one of a number of possible goods that we ought to balance against each other to bring about the best possible outcome. While it seems likely that reciprocity will contribute to overall good, it is hard to imagine an account focused only on reciprocity as the single good (whether or not to be maximized) as being remotely plausible. Other things are equally or more important, such as justice. However, an alternative extrinsic consequentialist account of reciprocity may seem more plausible. On this view reciprocity is something valued because of the role it has as a means to the ultimate good (e.g., utility, welfare, or whatever). On this view, reciprocity is an important component of what it is that makes our lives go well. It should be noted that such a view has some similarities with Becker’s account of reciprocity, and indeed, one version of such an extrinsic consequentialist account might see reciprocity as a disposition or virtue, likely to contribute towards human and social flourishing. Reciprocity on these extrinsic consequentialist accounts will be an important moral consideration. Part of what it is to be human is to live with others, and the everyday reciprocal relations between social beings are likely to contribute towards the realization of our ultimate ends.

How might a consequentialist account of reciprocity apply to TB treatment and control? First, isolation is something that can be justified using consequentialist reasoning. However, a focus on outcomes does not mean that individuals need to be sacrificed for the public good. A plausible pluralistic, extrinsic account of consequentialism can easily accommodate the idea that protection from harm, individual and public benefit, as well as justice might all be important reasons for actions, and might all be important aspects to weigh against each other in reaching a decision about what to do. Reciprocity is relevant here, as it seems plausible to see that it is in the long-term interest of public participation in public health programs, if those participating in isolation are cared for, helped or even compensated in appropriate ways. Indeed, on this approach, it may make a great deal of sense to take the idea of robust compensation for time spent in isolation, and other losses, very seriously indeed.

Second, in relation to novel TB drugs, likewise, there is no need to see a consequentialist approach as necessarily generating the easy answer of encouraging (or even forcing) treatment. It is true that some aggregative accounts that seek to maximize utility may argue in favour of forcing treatment on individuals for the good of others. However, on the pluralistic, extrinsic consequentialist view outlined above, the most effective outcome is to be balanced against other considerations, such as the long term damage to public health that might result from such action. However, if individuals agree to take such drugs, when they are used as a last resort and the individuals are informed, this type of consequentialism can again see the benefits that arise from such action, and appeal to reciprocity as a means to assist, compensate or even reward the actions of those with TB where they have risked their health, and thereby benefitted the community by reducing the risk of transmitting TB in addition to any individual benefits in relation to their own health.

Third, a pluralistic, extrinsic consequentialism may well see justice-based considerations, such as equity, as being an important element contributing to the overall chosen end. This means that such an account may see prevention of harm produced through injustice as something to motivate action. Where individuals or sub-groups in a population run greater risks of harm, reciprocity may require action to protect, compensate or reward, where others in the population benefit. As we will see below, such claims are related to justice but they are a separate and more specific claim.

## Section 3: Reciprocity and the Ethics of Infectious Diseases

As we have seen above, all three theoretical positions that we have considered can place a strong reliance on reciprocity, even if they use different means of justification and provide different degrees of application in the context of tuberculosis. In this section we sketch out the different elements that seem to be required for an account of reciprocity, defend the idea of reciprocity as a normative principle from some objections, and note how reciprocity can be distinguished from other normative considerations.

When evaluating the literature on the place of reciprocity in public health ethics, one thing that is striking is how most accounts do not clearly define what reciprocity is and how it is supposed to contribute to normative decision-making. Perhaps the best articulated account of reciprocity in the public health ethics literature is that of Viens and colleagues, which does provides a definition of reciprocity. They suggest that:

Reciprocity demands an appropriate balancing of the benefits and burdens of social cooperation necessary to obtain the good of public health. … Reciprocity requires that we compensate those disproportionately burdened by complying with restrictive measures and make restitution to those individuals wronged by being subjected to unfair or intolerable treatment. Reciprocity not only requires that individuals should not be overly burdened by measures to protect public health, but also that individuals are supported in a way that allows them to fulfil their obligations (Viens, Bensimon, and Upshur 2009, 211−212).

Building upon this definition, we believe we need a more general and formal articulation. A preliminary and atheoretical account of reciprocity is needed to link to the different theoretical accounts as outlined above, provide a clear idea of what is meant by the concept, and to begin concrete discussion about its application.

We might adapt Viens et al., and take from the spirit of the three more theoretical accounts in the previous section, to stipulate a definition for reciprocity as a mid-level public health ethics principle as follows: reciprocity is a moral obligation involving an appropriate response by *B* commensurate with an action by *A*, where *A’s* action aims to contribute to, or bring about, public good, and where the action involves burdens, costs, or risks of harm to *A*. This stipulation makes a number of key features of reciprocity clear. First, *A’s* action must be directed in the right way, namely, primarily towards public good and not other considerations such as personal benefit. Second, *B* has an obligation to act in response to *A’s* action. Third, *B’s* response must be appropriate and commensurate. In some cases this might involve strict compensation, but in other cases it might involve greater payment (e.g., punitive damages, rewards) than that strictly required by mere compensation. This may encompass cases such as isolation for life, where the loss is not merely a temporary one, or possibly cases where *A’s* original loss is ultimately held to be morally unjustified (e.g., if a person is found to have been ordered to maintain total physical isolation when maintaining respiratory isolation, by using a mask, was sufficient). Critically, this definition does not specify the motivation for such a principle. In other words, such a principle of reciprocity can be justified on the grounds of the direct goods and harms that befall persons (to appeal to those partial to Becker or a consequentialist account) or can be defended as a means of practically promoting a formal egalitarian account of self-mastery through compensation or reward (such that it may appeal to Kantians).

However, it is important to see that a principle of reciprocity can also entail much more than retrospective compensation. It is more than a “thank you” for a sacrifice, as in the case of acting gratefully toward healthcare workers who place themselves in danger (Silva and Viens 2015). For example, in all three accounts of reciprocity outlined in Section 2, we saw, to different degrees, that justice considerations may also feature appeals to reciprocity. In the context of TB, then, reciprocity forces us to acknowledge not just the immediate threats to the interests, welfare, or rights of those who have to abide by isolation orders, but we can also see reciprocity as a means of society and the state accepting responsibility for the conditions that have led to infection and disease from TB. In other words, if as noted above, TB is a social disease and a disease of poverty, part of the reason that the state has an obligation to support and compensate those in isolation from TB stems from this fact, namely that the transmission and burden of TB is disproportionately distributed and is (at least partly) caused by the moral failings of the state and society to eliminate poverty; to borrow from Becker, reciprocity here is compensation or restitution for harms committed.

We find such an account of reciprocity to be compelling. However, we should note that not all will be convinced. For example, Holm considers two objections to the claim of reciprocity for those who remain in isolation that are worth noting: responsibility for being infected and whether providing compensation can undermine the public good. First, one might argue that if *A* has a causal role in being infected with pathogen *B*, then *A* bears some of the responsibility to protect others, irrespective of state support, because they are responsible for being infected. Holm counters that although there may be some causal story to tell about how someone becomes infected, “it is implausible to claim that they were the causes of their own risk of infection in any morally interesting way” (Holm 2009, 200). TB is a paradigmatic example of Holm’s conclusion: as noted above, TB is a social disease and one borne from poverty, and as such, it is difficult to lay moral blame upon persons for becoming infected in the course of their daily lives (e.g., working in a factory, taking the bus to work, etc.).

Second, one might object that providing compensation and support to those who are, or have been, in isolation for an infectious disease might cost too much money, more than states can reasonably allocate, thereby causing more net-harm to society. Here Holm draws the distinction between moral reasons and practical reasons toward or away from a particular act. It might be true that there are important policy questions that must be asked and answered before implementing governmental changes, “but this does nothing to show that society does not have a strong obligation to compensate in those cases where it is clearly possible” (Holm 2009, 204). While the state can provide some support to persons in isolation with TB, it may very well be the case that the state cannot compensate them fully (especially in situations of isolation until death where one might argue any reward or compensation will necessarily be incommensurate to the loss). Moreover, at an international level, if a country curtails the spread of an infectious disease such that it has a positive effect of protecting other countries from being affected, then other states might hold some obligations to support those states most gravely affected (Silva and Smith 2015). Moreover, the obligation for states not affected by TB to support the states and the residents of those states most greatly affected might stem from the fact that those states generally not affected by TB, namely, high-income countries, often derive material benefits from the conditions that allow TB to flourish in low-and middle-income countries. For example, high-income countries derive benefits from the mining that occurs in South Africa, where housing for miners serves as a primary environmental contributor to the spread of TB.

A final note: although we cannot enter into a full discussion here, we acknowledge that greater consideration ought to be given to the role of reciprocity in the context of, and in contrast to, other public health values and norms, such as justice or equality. Indeed, Becker situates reciprocity in the broader context of morality from a virtue ethics viewpoint; while for Kant and Kantians, reciprocity is a key concept that helps structure and animates an equal notion of freedom; and for extrinsic, pluralist consequentialists, reciprocity can be a key component of a sophisticated and balanced account of relevant outcomes. For many theorists and theories, reciprocity will be only *a*, not *the*, key idea or principle within a broader moral and political theory of the right or the good. In the context of TB, many normative challenges arise, and many values, norms, and principles are at play. Reciprocity is not a free-standing principle and ought to be considered more closely in light of these other concepts within the realm of public health. For example, we have not delved into the types of harm that occur when a person with TB is placed in isolation. Perhaps whether one understands harm as physical, mental, or a loss of freedom, for example, will alter how one understands, applies, and justifies the use of reciprocity beyond that of a mid-level principle. Moreover, it is important to note that reciprocity cannot simply be reduced to other hereto established and accepted principles of bioethics or public health ethics. Although more can and ought to be said on this point, ideas such as beneficence (i.e., acting so as to promote the good of others), justice (i.e., in this context, distributing what is deserved), and solidarity (i.e., a commitment to care for others often in positions of vulnerability or marginalization) are different from reciprocity although there may be some overlap and reinforcement of key concepts within these other values. Clearly, further nuanced analysis will help develop our understanding of the concept of reciprocity and this may in turn help ground a general account of reciprocity in public health ethics.

## Conclusion

Tuberculosis is a disease of poverty, whereby persons of lower SES are not only at risk of contracting the pathogen and developing the disease but as a result are thereby subject to the clinical treatment and public health measures that risk their freedoms and cause them potential harm. In this paper, we have argued that reciprocity can be grounded via different normative theories, but that the result in practice is generally the same: that under a principle of reciprocity, not only must compensation and support be provided to those TB patients who abide by public health measures but also, in contrast to existing discussions of reciprocity in the public health ethics literature, that reciprocity may provide further arguments in favour of the moral obligation to rectify the background conditions that lead to contracting and developing TB in the first place.

## Footnotes

1 For this paper, we draw no distinction between *morality* and *ethics*, since (a) it does not seem to provide any further conceptual clarity to the arguments herein, and (b) at least etymologically, *moralis* was used as the Latin translation for the Greek word *ethikos*.

2 This raises a host of important questions for public health to consider, e.g., at what point does the probability of harm-to-self allow an individual to forgo a public health measure? Who makes such determinations? What if the risk must be taken to confer a public protection? Since isolation is generally sufficient to protect others from risk of contracting the TB bacteria and the risk of severe harm from new anti-tubercular drugs high, we forgo these questions here, though note that they ought to be addressed.
